# Antitumor efficacy of 5-aminolevulinic acid (5-ALA)-based radiodynamic therapy under single-dose X-ray irradiation in colon cancer

**DOI:** 10.3389/fonc.2025.1722919

**Published:** 2025-12-01

**Authors:** Junko Takahashi, Shinsuke Nagasawa, Xiupeng Wang

**Affiliations:** 1 Graduate School of Information, Production and Systems, Waseda University, Kitakyushu, Japan; 2 Department of Radiology, Graduate School of Medical Science, Kyoto Prefectural University of Medicine, Kyoto, Japan; 3 Health and Medical Research Institute, National Institute of Advanced Industrial Science and Technology (AIST), Tsukuba, Japan

**Keywords:** radiotherapy, radiodynamic therapy, single-dose irradiation, hypofractionatedirradiation, photodynamic diagnosis, 5-aminolevulinic acid, protoporphyrin IX, colorectal cancer

## Abstract

**Introduction:**

5-Aminolevulinic acid (5-ALA)-based radiodynamic therapy (RDT), an experimental approach that combines systemic administration of 5-ALA with ionizing radiation, has demonstrated antitumor efficacy primarily in preclinical studies using fractionated irradiation protocols. In recent years, single-dose and hypofractionated irradiation regimens have been increasingly adopted in clinical radiotherapy; however, the therapeutic potential of 5-ALA-based RDT under single-dose irradiation conditions remains to be elucidated.

**Methods:**

We evaluated the accumulation of protoporphyrin IX (PpIX) in human colon cancer HT-29 cells compared with previously studied cell lines, mouse melanoma B16/BL6 and human glioblastoma U-251 MG, U-87 MG *in vitro*. Using a HT-29 xenograft mouse model, we investigated the antitumor efficacy of a single 12 Gy X-ray dose combined with 5-ALA at doses of 100 or 200 mg/kg. Tumor growth, histopathological alterations, and immune cell infiltration were analyzed. Gene expression profiles of tumor tissues were examined by microarray analysis at day 29 post-irradiation.

**Results:**

HT-29 cells exhibited equal or greater PpIX accumulation compared with other tumor cell lines. *In vivo*, single-dose X-ray irradiation (12 Gy) combined with various doses of 5-ALA resulted in 5-ALA dose-dependent suppression of tumor growth. Notably, administration of 200 mg/kg 5-ALA plus 12 Gy X-ray induced marked tumor regression in all animals without statistically significant weight loss. Histopathological analysis demonstrated disruption of tumor cell islands and increased infiltration and proximity of Iba1-positive immune cells to tumor cells. Microarray analysis identified 75 differentially expressed genes between untreated and 200 mg/kg 5-ALA plus X-ray groups, including downregulation of genes involved in DNA repair, tumor suppression, autophagy, cell cycle regulation, metabolism, and immune evasion.

**Conclusion:**

This study demonstrates for the first time that 5-ALA combined with single-dose X-ray irradiation exerts a strong antitumor effect on HT-29 xenografts. The observed effects may be mediated by induction of immunogenic cell death, modulation of the tumor microenvironment, and suppression of tumor cell survival pathways. These findings highlight single-dose 5-ALA-based RDT as a potential novel therapeutic strategy.

## 1 Introduction

Radiation therapy is one of the major modalities for cancer treatment and is routinely applied to a wide range of solid tumors. However, conventional radiotherapy alone is often insufficient to achieve satisfactory therapeutic outcomes, and the development of more effective treatment strategies is urgently needed. Radiodynamic therapy (RDT) is an emerging approach that utilizes radiosensitizers capable of enhancing the generation of reactive oxygen species (ROS) through physicochemical interactions with X-rays. Previous studies have investigated low-molecular-weight organic compounds, such as protoporphyrin IX (PpIX), which function as radiosensitizers by producing hydroxyl radicals, singlet oxygen, and superoxide upon X-ray irradiation (1, 2).

5-Aminolevulinic acid (5-ALA), a precursor of PpIX, exhibits tumor-selective accumulation and is therefore expected to provide unprecedented radiosensitization at the cellular level. We refer to the combination of systemic 5-ALA administration and X-ray irradiation as 5-ALA-based radio-dynamic therapy (hereafter 5-ALA-based RDT). Clinical studies evaluating 5-ALA-based RDT are now underway (3, 4). Although several reviews have summarized the current understanding of RDT, the efficacy of 5-ALA-based RDT has mainly been examined in preclinical *in vivo* studies using fractionated irradiation protocols (5–7). In contrast, the potential effectiveness of single-fraction irradiation has not been sufficiently evaluated, leaving a gap in evidence relevant to single-fraction protocols in clinical settings.

Therefore, the present study was designed to evaluate the efficacy of 5-ALA–based RDT under single-fraction irradiation. A dose of 12 Gy delivered in a single fraction (12 Gy/1 fr) was selected as an experimental setting, representing a relatively higher level compared with clinically common single-fraction doses such as 8 Gy/1 fr or 10 Gy/1 fr. By focusing on single-fraction protocols, this study aims to provide new insights that may broaden current radiotherapeutic strategies beyond conventional fractionated irradiation.

## 2 Materials and methods

### 2.1 Cell culture

HT-29 human colon cancer cells (HT-29-Luc) and U-251MG human glioblastoma cells (U-251 MG-Luc) were obtained from the Japanese Collection of Research Bioresources (Osaka, Japan). Human arterial endothelial cells (HAECs) were obtained from KAC Co., Ltd. (Kyoto, Japan). The B16-BL6 mouse melanoma cell line was supplied by the RIKEN Cell Bank (Tsukuba, Japan). U-87 MG human glioblastoma cells were obtained from the American Type Culture Collection (ATCC, Manassas, VA, USA).

HT-29 and B16/BL6 cells were cultured in RPMI-1640 medium (FUJIFILM Wako, Osaka, Japan). U-251 MG and U-87 MG cells were cultured in Eagle’s minimum essential medium (EMEM; FUJIFILM Wako) supplemented with 10% fetal bovine serum (FBS; FUJIFILM Wako). The media were supplemented with 100 U/mL penicillin and 100 µg/mL streptomycin (FUJIFILM Wako). HAECs were cultured in endothelial cell growth medium-2 (EGM-2, Lonza, Basel, Switzerland) according to the manufacturer’s instructions. All cells were maintained in a humidified incubator at 37°C under 5% CO_2_.

### 2.2 Determination of PpIX concentration in cells

HT-29, HAECs, B16/BL6, U-251 MG, and U-87 MG cells were seeded in 25 cm² flasks and cultured at 37°C for 24 h. After 24 h of culture, the cells were incubated with 0, 0.5, or 1 mM 5-ALA (FUJIFILM Wako, Osaka, Japan) for 4 h.

Cells were washed twice with PBS and then lysed with 50 µL of 0.1 M NaOH containing 0.1% Triton X-100. An aliquot (10 µL) was used for protein concentration measurement using a modified Lowry protein assay kit (Thermo Scientific, Rockford, IL, USA). The remaining 40 µL of lysate was mixed with 160 µL of N,N-dimethylformamide:isopropanol (100:1, v/v) for protein denaturation. After overnight incubation, samples were centrifuged at 12,000 rpm for 10 min. Porphyrin concentrations were determined by spectrophotometry at 405 nm (Soret maximum), and fluorescence was measured with excitation at 405 nm and emission at 635 nm using a microplate reader (Synergy H1, Agilent Technologies, Santa Clara, CA, USA).

### 2.3 Animals and X-ray irradiation

Six-week-old female nude mice (BALB/c nu/nu; Charles River Laboratories Japan, Inc., Yokohama, Japan) were anesthetized by intraperitoneal administration of pentobarbital sodium (10–15 mg/kg) followed by inhalation of 2% isoflurane using a precision vaporizer. Subsequently, 2.0 × 10^6^ HT-29 cells suspended in 100 µL of PBS were subcutaneously injected into the right hindlimb.

When the tumor volume reached approximately 100 mm³, the mice were randomized into five groups to ensure uniform tumor size:(1) NT, control group (n = 4); (2) ALAT, 200 mg/kg 5-ALA administered orally (n = 4); (3) XT, 12 Gy X-ray irradiation (single fraction; n = 5); (4) ALA100-XT, 100 mg/kg 5-ALA administered orally 4 h before irradiation (n = 5); and (5) ALA200-XT, 200 mg/kg 5-ALA administered orally 4 h before irradiation (n = 5). 5-ALA was administered by oral gavage to ensure accurate dosing and to mirror the clinical route of administration. Clinically, 5-ALA is given orally at 20 mg/kg 2–4 hours before anaesthesia or surgery according to the European product label (8). The doses used in this study (100 and 200 mg/kg in mice) were determined based on body surface area conversion from the clinical human dose. In mice, oral administration yields reliable systemic absorption: intestinal permeability and oral pharmacokinetics are substantial and largely mediated by the proton-coupled oligopeptide transporter PEPT1, with dose-proportional systemic exposure after oral dosing (9).

X-ray irradiation was performed using an MBR-1520R-4 irradiator (Hitachi Power Solutions, Hitachi, Japan) at 150 kV and 20 mA with added filtration of 0.5 mm Al and 0.1 mm Cu. The dose rate was 1.0 Gy/min at the sample stage. For irradiation, each mouse was immobilized in a plastic holder with an opening above the tumor area. A collimated X-ray beam (20 × 20 mm field) with a lead plate was used to irradiate the tumor-bearing hindlimb, which fully encompassed the entire tumor region.

Mice in the 5-ALA plus X-ray treatment groups received oral administration of 5-ALA (diluted in PBS) at 0, 100, or 200 mg/kg body weight 4 h prior to X-ray irradiation. Mice in the 5-ALA-only groups received 0 or 200 mg/kg 5-ALA orally at the corresponding time points without irradiation. Tumor volume was measured weekly with a caliper and calculated according to the formula: Tumor volume = (shortest diameter)^2^ × (largest diameter) ×0.5.

Mice in non-irradiated groups were sacrificed when tumor volume reached 1000 mm³. Tumors were weighed using an electronic balance. All mice were euthanized 29 days after irradiation by intraperitoneal injection of an overdose of pentobarbital sodium (150–200 mg/kg) followed by cervical dislocation to ensure death, according to the AVMA Guidelines for the Euthanasia of Animals (2020).

### 2.4 Immunocytochemistry and morphological observation of tumor tissues

Tumor tissues were fixed in 4% paraformaldehyde and embedded in paraffin. Sections (6 µm) were deparaffinized in xylene and rehydrated through graded ethanol. Morphology was assessed with hematoxylin and eosin (H&E) staining.

Antigen retrieval was performed in 10 mmol/L EDTA buffer (pH 8.0) at 92°C for 10 min. Endogenous peroxidase was blocked with 0.3% H_2_O_2_ for 12 min. Non-specific binding was blocked with 5% goat serum for 30 min at 37°C. Sections were incubated overnight at 4°C with anti-Iba1 primary antibody (rabbit polyclonal, 1:1000 dilution; FUJIFILM Wako, Osaka, Japan, Cat# 019-19741, validated for immunohistochemistry in mouse tissues), followed by 30 min incubation at 37°C with Polink-1 HRP Rabbit DAB kit (18 mL; Cosmo Bio, Tokyo, Japan) according to the manufacturer’s instructions. Antigen–antibody complexes were visualized using DAB, and sections were counterstained with Mayer’s hematoxylin solution (FUJIFILM Wako) for 20 s at room temperature.

### 2.5 Microarray analysis

Tumor samples for microarray analysis were collected in RNAlater (Qiagen GmbH, Hilden, Germany), incubated at 4°C overnight to stabilize RNA, and then stored at −80°C. Total RNA was extracted from tumor tissues using the RNeasy Mini Kit (Qiagen GmbH) following the manufacturer’s instructions. RNA quality was assessed using an Agilent 2100 Bioanalyzer (Agilent Technologies, Santa Clara, CA, USA), and RNA concentration was measured with a NanoDrop ND-1000 spectrophotometer (NanoDrop Technologies, Wilmington, DE, USA).

Cyanine 3-CTP-labeled cRNA was hybridized to human oligo microarray slides (#G4112F Whole Human Genome Microarray (44K), Agilent Technologies) at 65°C for 17 h. Slides were washed according to the manufacturer’s protocol and scanned with an Agilent DNA Microarray Scanner (#G2565BA, Agilent Technologies) at 5 µm resolution. Images were analyzed using Agilent Feature Extraction Software version 9.5.3.1. Data were normalized by quantile normalization. Differential expression analysis was performed using the limma package (version 3.64.3) in R (version 4.3.1; R Core Team). Genes with FDR < 0.05 and fold change ≥ 1.5 or ≤ 0.67 were considered significant. Some genes with fold changes slightly below these thresholds (e.g., ratio ≈ 1.4) were also detected due to statistical significance. Microarray experiments were MIAME compliant, and raw data were deposited in GEO (Accession number GSE308518).

(http://www.ncbi.nlm.nih.gov/geo/query/acc.cgi?acc=GSE308518)

### 2.6 Statistical analysis

Statistical comparisons among groups were performed using one-way ANOVA. Homogeneity of variance was assessed with Bartlett’s test. When variances were equal (p ≥ 0.05), *post hoc* comparisons were performed using Tukey’s honestly significant difference test. When variances were unequal (p < 0.05), the Games–Howell test was applied. Differences were considered statistically significant at p < 0.05. Correlations in gene expression were assessed using Pearson’s correlation coefficients, and Fisher’s Z-transformation was applied to approximate a normal distribution. All statistical analyses were performed using R software (version 4.3.1; R Foundation for Statistical Computing, Vienna, Austria) within the RStudio environment (version 2025.09.0; Posit Software, Boston, MA, USA).

### 2.7 Ethical considerations

All experimental protocols were approved by the Committee for the Care and Use of Experimental Animals at the National Institute of Advanced Industrial Science & Technology (AIST) (permit number 2020-097). All efforts were made to minimize animal suffering and the number of animals used.

## 3 Results

### 3.1 PpIX accumulation

**Figure 1:**
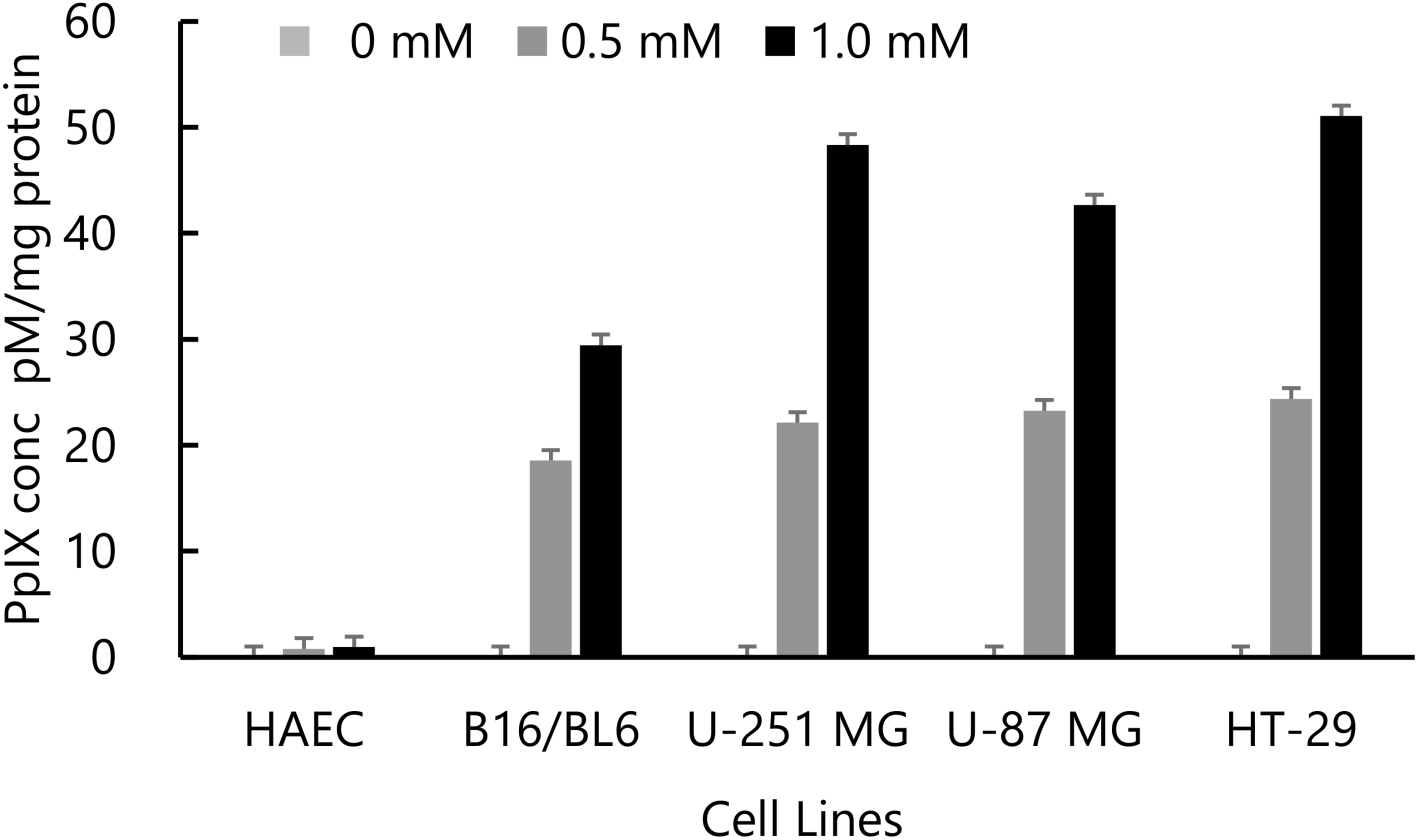
PpIX accumulation was measured in human arterial endothelial cells (HAECs), B16/BL6 mouse melanoma cells, U-251 MG and U-87 MG human glioma cells, and HT-29 colorectal adenocarcinoma cells after a 4 h exposure to 0, 0.5, and 1 mM 5-aminolevulinic acid (5-ALA).

Prior to the *in vivo* experiments, intracellular accumulation of PpIX was examined in various cell lines *in vitro*. We have previously evaluated the efficacy of 5-ALA–based RDT against melanoma and glioma. RDT is based on the physicochemical reaction between X-rays and PpIX accumulated within tumor cells. Therefore, we compared the intracellular PpIX accumulation in the previously reported cell lines (10, 11) with that in HT-29 cells *in vitro* (**Figure 1**). As a result, PpIX accumulation in HT-29 cells was comparable to or greater than that observed in B16/BL6, U-251 MG, and U-87 MG cells, suggesting that 5-ALA–based RDT may also be effective in HT-29 cells.

### 3.2 Combination of 5-ALA and single dose X-ray irradiation suppresses tumor growth *in vivo*

Most of the previously reported efficacies of 5-ALA–based RDT have been obtained using fractionated low-dose irradiation protocols, whereas single-dose irradiation has often failed to produce comparable effects (12–14). In clinical photodynamic diagnosis (PDD), the human dose corresponds to approximately 240 mg/kg when converted to a mouse equivalent dose. Because fractionated irradiation allows repeated dosing, lower 5-ALA concentrations are frequently employed in those settings (6). In contrast, single-dose irradiation is more challenging. Therefore, in this study, we evaluated the efficacy of 5-ALA-based RDT using a dose of 100 mg/kg (previously shown to be effective in glioma) and a higher dose of 200 mg/kg 5-ALA.

**Figure 2:**
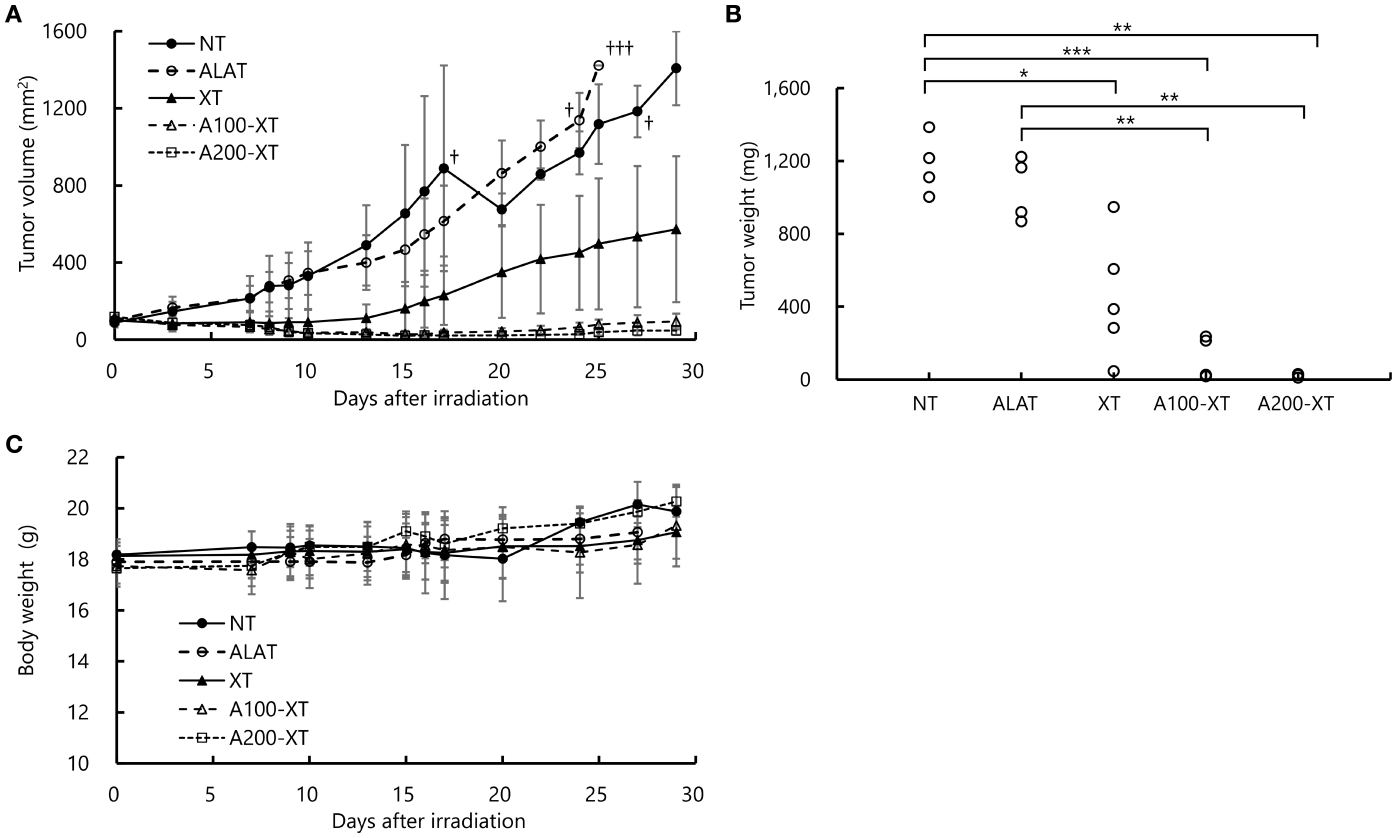
*In vivo* antitumor effects of 5-ALA combined with single-dose X-ray irradiation. **(A)** Tumor growth curves of HT-29 xenografts under different treatment conditions: no treatment (NT), 5-ALA alone (ALAT; 200 mg/kg), X-ray alone (XT; 12 Gy), 5-ALA 100 mg/kg + X-ray (A100-XT), or 5-ALA 200 mg/kg + X-ray (A200-XT). Tumor volumes were monitored for 29 days. In the NT and ALAT groups, some tumors exceeded the humane endpoint threshold (>1000 mm³), and these animals were euthanized (†). The † symbol indicates animals that were euthanized upon reaching the humane endpoint. **(B)** Final tumor weights of HT-29 xenografts at the end of the experiment. Mean tumor weights were as follows: NT, 1178.4 ± 163.1 mg; ALAT, 1043.0 ± 175.8 mg; XT, 453.4 ± 293.6 mg (showing variable tumor suppression); A100-XT, 101.9 ± 96.9 mg; and A200-XT, 23.5 ± 8.9 mg. Both A100-XT and A200-XT exhibited marked and dose-dependent tumor growth inhibition compared with NT and ALAT groups. **(C)** Body weight changes during the experimental period. No significant body weight loss was observed in any treatment group, indicating that the treatments were well tolerated without overt systemic toxicity. *p < 0.05, **p < 0.01, ***p < 0.001.

Tumor volume over time, tumor weight at necropsy, and body weight changes are shown in **Figures 2A–C**. The observation period was 29 days. In the NT (no treatment) and ALA-T (5-ALA alone) groups, tumor volume continued to increase, and several mice exceeded the humane endpoint of 1000 mm² tumor volume and were euthanized accordingly. The XT (X-ray only) group exhibited slower tumor growth compared with the NT and ALA-T groups, although tumors continued to enlarge in most mice; spontaneous tumor regression was observed in some individuals. In the A100-XT group (100 mg/kg 5-ALA + X-ray), tumor growth was further suppressed, whereas in the A200-XT group (200 mg/kg 5-ALA + X-ray), marked inhibition and even regression of tumor growth were observed (**Figure 2A**).

At the end of the experiment, mean tumor weights were 453.4 ± 293.6 mg in the XT group, 101.9 ± 96.9 mg in the A100-XT group, and 23.5 ± 8.9 mg in the A200-XT group, indicating a dose-dependent enhancement of tumor growth suppression and regression with increasing 5-ALA concentrations (**Figure 2B**). No significant changes in body weight were observed in any group during the experimental period (**Figure 2C**).

### 3.3 Histopathological observation of tumor tissues

**Figure 3:**
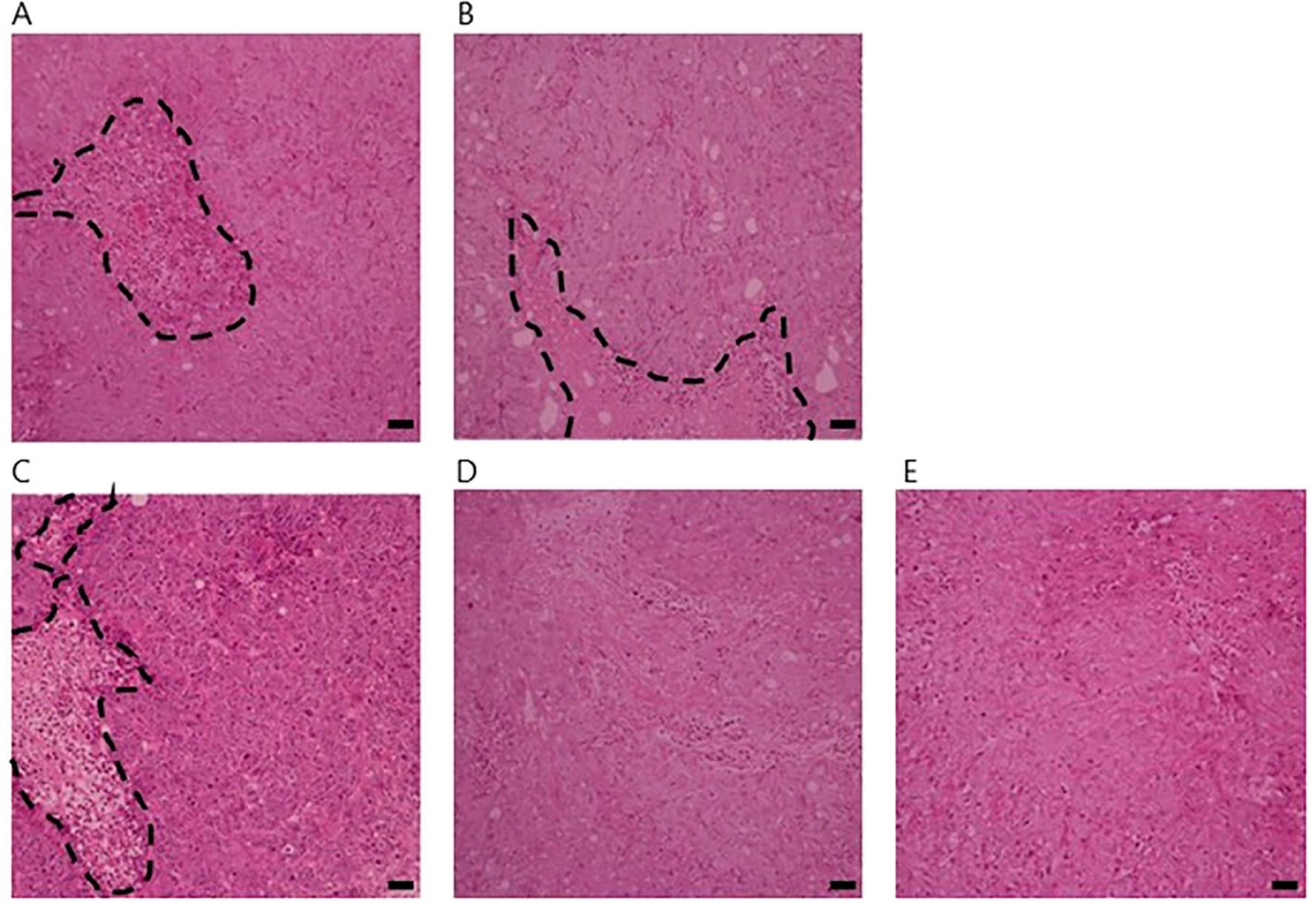
Histopathological analysis of HT-29 xenograft tumors (HE staining). Histopathological analysis of HT-29 xenograft tumors by hematoxylin and eosin (HE) staining. Representative tumor sections are shown for the following groups: **(A)** NT, **(B)** ALAT, **(C)** XT, **(D)** A100-XT, and **(E)** A200-XT. NT and ALAT tumors exhibited stromal components of murine origin forming island-like structures within the tumor tissue. HT-29 cells displayed morphological features consistent with well-differentiated adenocarcinoma, including relatively dispersed chromatin and uniformly pale-staining nuclei. In contrast, tumors from the XT, A100-XT, and A200-XT groups demonstrated altered architecture following treatment, with loss of distinct glandular or stromal patterns. Black dashed lines indicate the boundary between tumor and stroma. Scale bar, 50 μm.

In hematoxylin and eosin (H&E)-stained tumor sections, the NT (no treatment) and ALA-T (200 mg/kg 5-ALA alone) groups showed distinct islands of tumor cells surrounded by scattered murine stromal elements. The nuclei of HT-29 cells displayed features consistent with well-differentiated adenocarcinoma, including relatively dispersed chromatin and uniformly pale nuclear staining (**Figure 3**).

**Figure 4:**
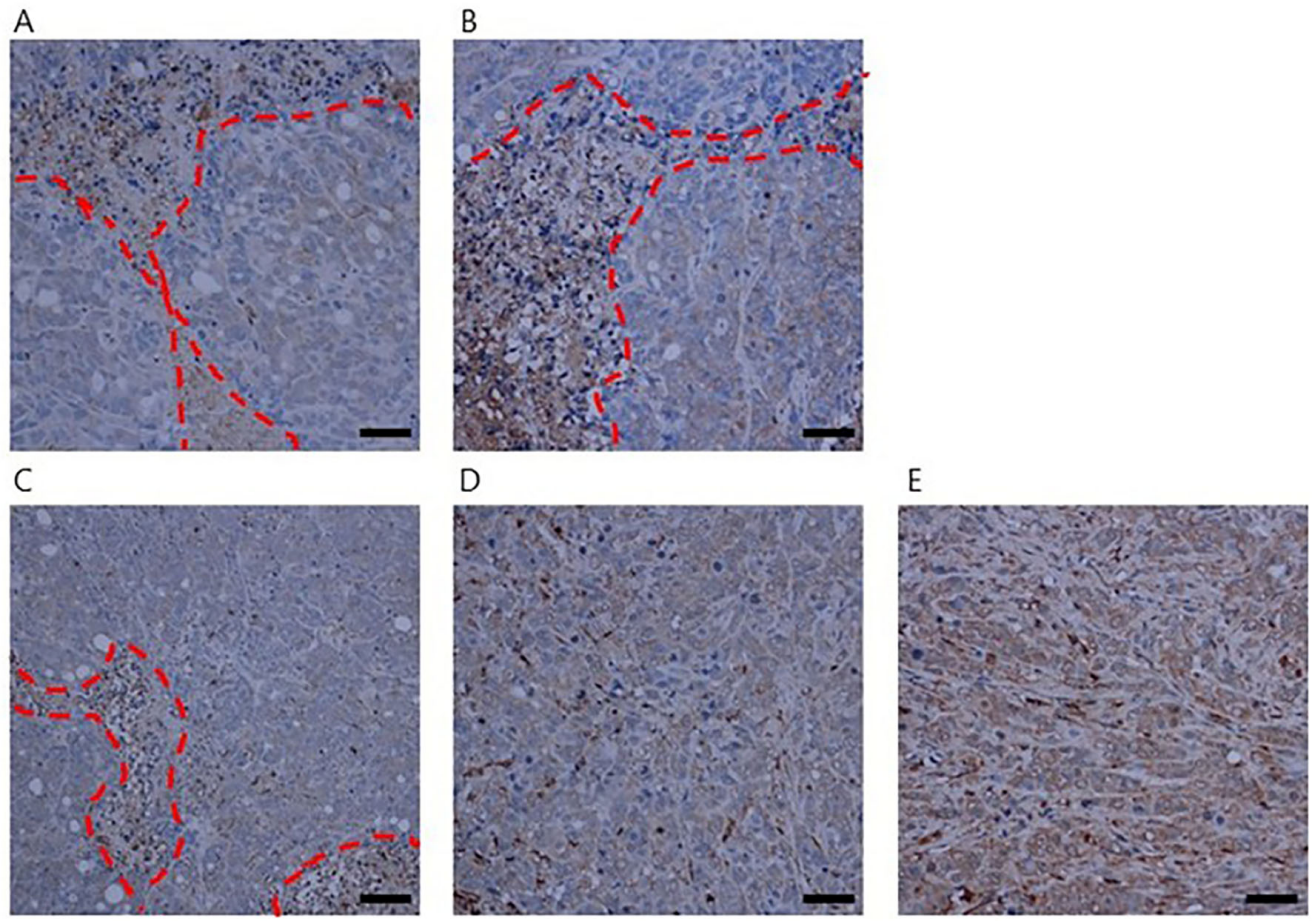
Immunohistochemical analysis of tumor-associated macrophages in HT-29 xenograft tumors using Iba1 staining. Representative tumor sections are shown for the following groups: **(A)** NT, **(B)** ALAT, **(C)** XT, **(D)** A100-XT, and **(E)** A200-XT. In NT and ALAT tumors, tumor cell islands were observed accompanied by scattered host-derived stromal components. In contrast, tumors from XT, A100-XT, and A200-XT groups exhibited disrupted architecture. Notably, in A100-XT and A200-XT tumors, close spatial association between tumor cells and host immune cells (macrophages) was frequently observed. Red dashed lines indicate the boundary between tumor cells and murine stromal components. Scale bar, 50 μm.

In contrast, the XT (X-ray only), A100-XT (100 mg/kg 5-ALA + X-ray), and A200-XT (200 mg/kg 5-ALA + X-ray) groups exhibited marked disruption of these island-like structures. Immunohistochemical staining with anti-mouse Iba1 antibody further highlighted these changes, particularly in the A100-XT and A200-XT groups, where numerous instances of proximity between tumor cells and murine immune cells were observed (**Figure 4**).

### 3.4 Analysis of microarray gene expression profiles

**Figure 5:**
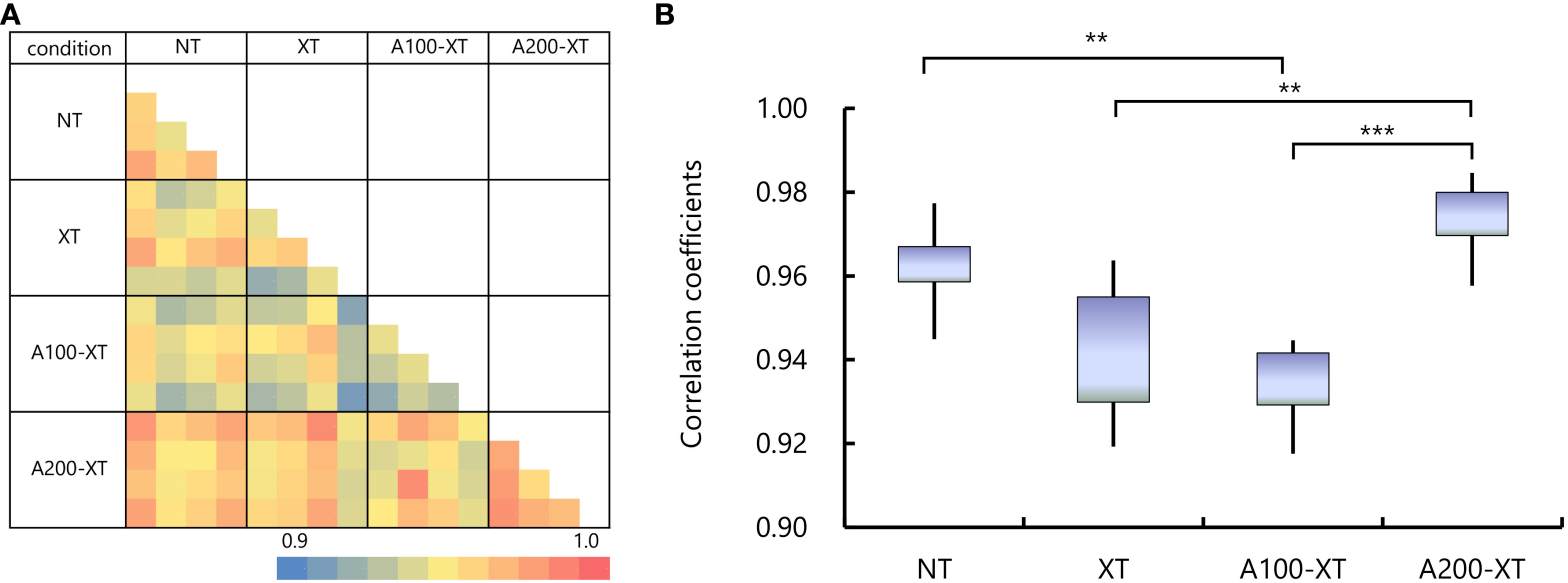
Microarray gene expression profiling of HT-29 xenograft tumor tissues following treatment. **(A)** Pairwise correlation matrix of normalized gene expression signals from tumor tissues collected 29 days after a single dose of 12 Gy X-ray irradiation. Samples included NT (no treatment), XT (X-ray only), A100-XT (5-ALA 100 mg/kg + X-ray), and A200-XT (5-ALA 200 mg/kg + X-ray) groups (n = 4 per group). Color intensity represents the Pearson’s correlation coefficient, reflecting the degree of similarity between individual microarrays. **(B)** Mean within-group Pearson correlation coefficients for each treatment group. The lower and upper boundaries of each box represent the 25^th^ and 75^th^ percentiles respectively. The lower and upper whiskers denote the minimum and maximum values, respectively. Data are presented as mean ± SD (n = 4; **p < 0.01, ***p < 0.001). Lower coefficients in the XT and A100-XT groups indicate increased inter-individual variability, whereas the higher coefficients observed in the A200-XT group indicate more homogeneous gene expression profiles among samples.

Comprehensive gene expression profiling was performed on tumor tissues collected 29 days after single-dose 12 Gy X-ray irradiation. Microarray analyses were conducted for NT, XT, A100-XT, and A200-XT groups (n = 4 per group). Pearson’s correlation coefficients were calculated from normalized signals for all 16 microarrays. A color-coded pairwise correlation matrix is shown in **Figure 5A**, where color intensity indicates the strength of the correlation. **Figure 5B** shows the mean within-group correlation coefficients, reflecting inter-individual variability in gene expression.

The mean (± SD) within-group correlation coefficients were 0.962 ± 0.011 for NT, 0.943 ± 0.018 for XT, 0.932 ± 0.010 for A100-XT, and 0.976 ± 0.010 for A200-XT. Correlation coefficients were lower in the XT and A100-XT groups but highest in the A200-XT group, consistent with the reduced tumor volumes and weights and lower variability observed in this group.

Between-group comparisons revealed high overall correlations between NT and A200-XT groups. However, differential expression analysis using FDR < 0.05 identified only one significantly altered gene in NT–XT and NT–A100-XT comparisons, whereas 75 genes (12 upregulated, 58 downregulated) were identified between NT and A200-XT (see **Supplementary Tables S1–S3**). Upregulated genes were primarily associated with cell adhesion/invasion/metastasis (ELMO2, ADAM9, DLG1, TRPM7), metabolism/mitochondrial function (FAM120A, DARS2), and cellular transport/axonal trafficking (KIF1B, TBC1D5), indicating the presence of tumor microenvironment stress. Downregulated genes included those related to DNA repair/tumor suppression (HTATIP, PDCD4, ERCC2), autophagy/protein degradation (FBXW9, TRIM63, CHMP6, TBC1D14), transcription factors/cell cycle regulation (CREB3L4, TLE3, ZNF226, ZNF629, TFDP2, ZNF496), cytoskeletal/signaling processes (ARHGAP4, KIF25, ARL16), metabolic pathways (PEX16, CKB, MT1F, MRPL21), and immune/cancer antigen-related functions (CCR3, PELI2, BTN2A2, CTAG1A). These changes suggest that tumor cells in the A200-XT group underwent metabolic stress, impaired stress-response capacity, and reduced proliferative potential.

## 4 Discussion

Radiotherapy protocols vary widely, encompassing conventional low-dose fractionation, hypofractionation, and single-dose irradiation. In recent years, hypofractionated and single-dose protocols have gained increasing attention worldwide. This trend has been facilitated by the widespread adoption of high-precision radiotherapy techniques, such as IMRT, SBRT, IGRT, and stereotactic irradiation, which allow higher doses per fraction to be delivered with reduced risk to normal tissues. In addition, reducing the number of treatment sessions improves patient quality of life and accessibility to therapy, and it has met an increased demand during the COVID-19 pandemic (15–17). Such technological advances have also encouraged exploration of higher single-fraction doses in various tumor settings.

Meanwhile, stereotactic radiosurgery (SRS) for liver metastases—mainly those originating from primary colorectal adenocarcinoma tumors—has been reported with doses ranging from 14 Gy to 26 Gy in a single fraction and 26 Gy to 60 Gy in three fractions (18). For brain metastases, postoperative SRS dose recommendations vary according to the cavity volume, and studies have investigated single-fraction doses between 12 Gy and 20 Gy (19). Based on these precedents, we investigated the potential of 5-ALA-based RDT in the context of both single- and hypofractionated irradiation.

Preclinical studies of 5-ALA-based RDT have primarily employed fractionated irradiation protocols. These studies consistently reported tumor growth inhibition and prolonged survival *in vivo* (10, 11, 13, 20–29). In contrast, few studies have evaluated single-dose irradiation *in vivo*, and their results have been inconsistent (12–14, 30, 31). To better understand these discrepancies, we summarized previous reports in **Table 1** (fractionated irradiation studies) and **Table 2** (single-dose irradiation studies). Among fractionated irradiation studies, only 2 out of 13 reported no significant efficacy (**Table 1**). For example, Dupin et al. employed IDH-wildtype GB patient-derived xenografts, which are presumed to exhibit high chemoradiotherapy resistance (26), and their irradiation schedule was alternate-day rather than consecutive, which may explain the reduced effect. Similarly, Viswanath et al. used only 8 Gy in two fractions, which might have been insufficient (29). Another important factor is the intrinsic PpIX accumulation capacity of cancer cells. While 5-ALA-induced PpIX accumulation is well characterized in cancers targeted by photodynamic diagnosis (PDD) or photodynamic therapy (PDT), less is known about its accumulation in deep-seated tumors that may benefit from RDT. Exogenous administration of 5-ALA initiates heme biosynthesis through the intrinsic cellular pathway. In cancer cells, accumulation of PpIX occurs due to reduced activity of ferrochelatase (FECH) and dysregulation of porphyrin transport systems (32, 33). Because the heme biosynthetic pathway takes place in mitochondria, PpIX predominantly localizes within this organelle at high concentrations. In PDT, light irradiation activates PpIX to generate ROS, whereas in PDD, PpIX emits fluorescence upon excitation, enabling visualization of tumor tissues (34). These mechanisms collectively explain the tumor-selective localization and diagnostic potential of 5-ALA-induced PpIX, providing the rationale for its application in RDT. Previous studies have compared PpIX accumulation among various cell lines (35), underscoring the need to consider this variable. In our study, HT-29 cells accumulated PpIX at levels comparable to or higher than those of B16/BL6, U251MG, and U87MG (**Figure 1**). Furthermore, Yamada et al. have already demonstrated the efficacy of 5-ALA-based RDT under a fractionated schedule of 15 Gy in five fractions (13), supporting that HT-29 cells possess sufficient metabolic activity for heme synthesis to accumulate PpIX.

**Table 1:** List of studies on 5-ALA-based RDT with fractionated irradiation *in vivo*.

Tumor entity	Cell line or type	5-ALA			Beam energy (kVp or MeV)	RT dose	Observed effect of 5-ALA + RT	Ref.
		Dose	Route	Timing (h prior to RT)				
Melanoma	B16-BL6	50 mg/kg	i.t.	24	100 kVp	30 Gy in 10 fx	Significant delay in tumor growth	(20)
Glioma	9L	100 mg/kg	i.v.	3	–	10 Gy in 5 fx	Significant delay in tumor growth	(21)
Glioma	MES-GSC	240 mg/kg	i.p.	–	–	30 Gy in 10 fx	Significant increase in median survival	(22)
Melanoma	B16-BL6	50 mg/kg	i.t.	24	100 kVp	30 Gy in 10 fx	Significant delay in tumor growth	(23)
Melanoma	B16-BL6	50 mg/kg	i.t.	4- 5	4 Mev 160 kVp	20 or 30 Gy in 10 fx	Significant delay in tumor growth	(10)
Melanoma	B16-Luc	200 mg/kg	i.p.	4	160 kVp	14 Gy in 7 fx	Significant delay in tumor growth	(24)
Prostate	MyC-CaP	30 mg/kg	p.o.	–	150 kVp	12 Gy in 3 fx	Significant delay in tumor growth	(25)
Colorectal	HT29	5 mg/mouse	i.p.	4	–	15 Gy in 5 fx	Significant delay in tumor growth	(13)
GBM	U251/U87	60 or 120 mg/kg	p.o.	4	150 kVp	60 Gy in 30 fx	Significant delay in tumor growth	(11)
GBM	Human GBM cells	100 mg/kg	i.p.	4	220 kVp	6 Gy in 3 fx, 10 Gy in 5 fx, 15 Gy in 5 fx	No enhancement of radiation efficiency	(26)
NSCLC	H1299	50 mg/kg	i.p.	4	320 kVp	15 Gy in 3 fx	Significant delay in tumor growth	(27)
Prostate	MyC-CaP	30 mg/kg	p.o.	3	150 kVp	20 Gy in 10 fx	Significant delay in tumor growth	(28)
Breast	4T1	5 mg/mouse	p.o.	4	320 kVp	8 Gy in 2 fx	No enhancement of radiation efficiency, but a significant delay in tumor growth was observed in combination with calcium tungstate nanoparticles and 5-ALA.	(29)

5-ALA, aminolevulinic acid; fx, fractions; GBM, glioblastoma; i.t., intratumoral; i.v., intravenous; i.p., intraperitoneal; p.o., per os (oral gavage).

**Table 2:** List of studies on 5-ALA-based RDT with single-fraction irradiation *in vivo.*

Tumor entity	Cell line or type	5-ALA			Beam Energy (kVp or MeV)	RT dose	Observed effect of 5-ALA + RT	Ref.
		Dose	Route	Timing (h prior to RT)				
Sarcoma	Lewis sarcoma	200 mg/kg	–	2	50 kVp	3 Gy in 1 fx	No enhancement of radiation efficiency, but a significant delay in tumor growth was observed with Photofrin.	(12)
Colorectal	HT29	5 mg/mouse	i.p.	4	–	1Gy in 1 fx	No enhancement of radiation efficiency	(13)
Prostate	PC−3	100 mg/kg	i.v.	4	15 MV	4 Gy in 1 fx	No enhancement of radiation efficiency, but a significant delay in tumor growth was observed in combination with carbamide peroxide and 5-ALA.	(14)
SCLC	KP1	100 mg/kg	i.v.	4	15 MV	4 Gy in 1 fx	Significant delay in tumor growth	(30)
GBM	HGG13	80 mg/kg	p.o.	26.5	1 MW Newtron	21.9 Gy Eq in 1 fx	Significant prolongation of survival	(31)

By contrast, among five single-dose irradiation studies, three reported no efficacy (**Table 2**). In all such cases, the delivered dose was ≤4 Gy, which is likely insufficient to elicit a robust effect (12–14). Interestingly, Penetta et al. and Yang et al. both used 4 Gy single-dose irradiation with a 15 MV Linac; they observed no effect in PC-3 prostate cancer cells but observed efficacy in KP1 small-cell lung cancer cells (14, 30). This suggests that, in addition to dose, the intrinsic properties of the cell line influence the outcome. Fukumura et al. employed boron neutron capture therapy (BNCT), with a BPA-derived Gy-equivalent dose of 21.9 Gy (31). Although this dose is theoretically sufficient, their single-dose 5-ALA-based RDT protocol involved a different beam modality. To date, no study has validated the efficacy of single-dose 5-ALA-based RDT under low-energy beam conditions. Thus, our present study is, to our knowledge, the first to demonstrate clear antitumor effects of single-dose 5-ALA-based RDT under such conditions.

The biological basis for fractionated irradiation efficacy is classically explained by the “4 Rs” of radiobiology (Repair, Redistribution, Repopulation, and Reoxygenation). In single-dose irradiation, these processes are largely absent. The antitumor effect of single-dose irradiation is thought to derive from direct tumor cell death combined with indirect mechanisms, such as vascular injury and immune activation (36–38). HT-29 tumors are known to harbor a complex tumor microenvironment (TME) with fibrotic stroma, cancer-associated fibroblasts (CAFs), and various immune cell populations (39–41). In our study, the combination of 5-ALA and X-ray treatment (particularly A200-XT) disrupted the island-like structure of tumor cells and increased the proximity of Iba1-positive immune cells to the tumor cells. These histological observations suggest that 5-ALA-based RDT may influence immune cell localization within the tumor microenvironment. Although this may indicate enhanced tumor cell recognition or activation of intratumoral immune cells, such mechanisms remain speculative because no flow cytometry, cytokine profiling, or immunophenotyping analyses were performed in this study. Recent studies have shown that both PDT and radiotherapy can induce immunogenic cell death and reshape the tumor immune microenvironment (42, 43), and our findings may be consistent with these processes; however, further studies are required to confirm whether immunogenic cell death directly contributes to the observed immune cell infiltration.

In this study, we demonstrated that a single 12 Gy dose combined with ≥100 mg/kg of 5-ALA produced a clear antitumor effect, with 200 mg/kg inducing tumor regression in all animals without evident toxicity. This finding supports the hypothesis that higher 5-ALA doses increase intratumoral PpIX production and radiosensitization, even under a single-dose regimen.

Microarray analysis revealed that, compared to XT and A100-XT groups, A200-XT exhibited higher intra-group correlation, consistent with the smaller variance in tumor volume and weight. In the NT–A200-XT comparison, 70 genes were significantly altered (FDR < 0.05), with most being downregulated genes related to DNA repair, tumor suppression, autophagy, cell cycle regulation, metabolism, membrane transport, and immune function. This suggests that the combined therapy impairs tumor cell survival strategies such as DNA damage response, metabolic maintenance, and immune function (including CCR3, SPNS3, SPPL3, and BTN2A2). Conversely, upregulated genes included those related to adhesion, invasion, and metabolic or transport processes (e.g., ADAM9), possibly reflecting a stress-induced attempt at metabolic reprogramming. These molecular signatures may serve as biomarkers for treatment response and provide insights into mechanisms of therapy resistance. Although the present study identified differentially expressed genes associated with 5-ALA–based RDT, these transcriptomic changes were not validated by quantitative PCR or protein-level analyses. Therefore, the results should be interpreted as preliminary molecular insights that warrant further experimental confirmation.

In summary, our study extends previous work by demonstrating that high-dose 5-ALA combined with single-dose X-ray irradiation achieves potent antitumor effects in a colorectal adenocarcinoma xenograft model. This effect appears to involve not only direct tumor cell damage but also alterations in the tumor microenvironment and transcriptional reprogramming. These findings provide a strong rationale for further investigation of 5-ALA-based RDT under hypofractionated or single-dose protocols, which may offer practical and clinical advantages over traditional fractionated regimens.

## 5 Conclusion

In this study, we demonstrated for the first time that 5-ALA combined with single-fraction X-ray irradiation exerts a remarkable tumor-suppressive effect in 5-ALA-based RDT, which has previously been reported primarily with fractionated irradiation. Notably, all animals in the 200 mg/kg 5-ALA group exhibited tumor regression without apparent toxicity. Histological and gene expression analyses may suggest that the observed effects involve the induction of immunogenic cell death, alterations in the tumor immune microenvironment, and suppression of DNA repair, metabolic, and immune evasion pathways. These findings support the possibility that 5-ALA-based RDT may be effective not only with fractionated but also with single-fraction irradiation, providing a foundation for future biomarker discovery and elucidation of mechanisms underlying treatment resistance.

## Data Availability

The raw data supporting the conclusions of this article will be made available by the authors without undue reservation. The microarray data have been deposited in the Gene Expression Omnibus (GEO) under accession number GSE308518. Additional supporting information is provided in the **Supplementary Material**.
