# Supplementary material for: Antitumor efficacy of 5-aminolevulinic acid (5-ALA)-based radiodynamic therapy under single-dose X-ray irradiation in colon cancer
Source: Front Oncol. 2025 Dec 1;15:1722919. doi: 10.3389/fonc.2025.1722919 (PMC12702768; doi:10.3389/fonc.2025.1722919)
Supplement: Supplementary file 1 [file Table1.docx]

Supplementary Material

Table S1 Differentially expressed genes identified by microarray analysis between NT and XT or A100-XT groups.

| Transcript ID | Gene symbol | FDR | Ratio | | |
| --- | --- | --- | --- | --- | --- |
|  |  |  | XT vs NT | A100-XT vs NT | A200-XT vs NT |
| NM_001837 | CCR3 | 0.012  (XT vs NT)  0.026  (A100-XT vs NT) | 0.2 | 0.2 | 0.2 |

List of genes differentially expressed (FDR-adjusted *p* < 0.05) in HT-29 xenograft tumors between the non-treated control (NT) group and either the X-ray–only (XT) or 5-ALA 100 mg/kg + X-ray (A100-XT) groups. Each group consisted of four independent samples (n = 4). Gene expression levels were analyzed by microarray followed by FDR correction using the *limma* package in R. Expression ratios were calculated as the mean signal intensity of the treated group divided by that of the control group (treated/control). Values > 1 indicate upregulation, and values < 1 indicate downregulation.

Table S2 Upregulated genes in HT-29 xenografts following 5-ALA (200 mg/kg) combined with X-ray irradiation.

| Transcript ID | Gene symbol | FDR | Ratio | | |
| --- | --- | --- | --- | --- | --- |
|  |  |  | XT vs NT | A100-XT vs NT | A200-XT vs NT |
| NM_001135920 | RIC1 | 0.034 | 1.3 | 1.4 | 2.4 |
| NM_014612 | FAM120A | 0.034 | 1.4 | 1.3 | 1.4 |
| NM_003455 | ZNF202 | 0.045 | 1.4 | 1.5 | 2.0 |
| NM_018122 | DARS2 | 0.045 | 1.2 | 1.5 | 2.0 |
| NM_020799 | STAMBPL1 | 0.045 | 1.5 | 1.7 | 2.0 |
| NM_001365953 | KIF1B | 0.047 | 1.4 | 1.3 | 1.9 |
| NM_182764 | ELMO2 | 0.047 | 2.7 | 2.4 | 2.9 |
| NM_003816 | ADAM9 | 0.049 | 1.2 | 1.5 | 1.8 |
| NM_004087 | DLG1 | 0.049 | 1.1 | 1.3 | 1.5 |
| NM_017672 | TRPM7 | 0.049 | 1.3 | 1.4 | 1.4 |
| NM_014744 | TBC1D5 | 0.050 | 0.9 | 1.2 | 1.4 |
| ENST00000507284 | FKBP4P1 | 0.050 | 1.7 | 1.9 | 2.3 |

List of genes significantly upregulated (FDR-adjusted *p* < 0.05) in tumors from the 5-ALA 200 mg/kg + X-ray (A200-XT) group compared with the non-treated control (NT) group (n = 4 per group). Differential expression was determined using the *limma* package after quantile normalization of microarray data.

Table S3. Downregulated genes in HT-29 xenografts following 5-ALA (200 mg/kg) combined with X-ray irradiation.

| Transcript ID | Gene symbol | FDR | Ratio | | |
| --- | --- | --- | --- | --- | --- |
|  |  |  | XT vs NT | A100-XT vs NT | A200-XT vs NT |
| NM_001837 | CCR3 | 0.011 | 0.2 | 0.2 | 0.2 |
| NM_004813 | PEX16 | 0.034 | 0.7 | 0.6 | 0.5 |
| NM_032301 | FBXW9 | 0.034 | 0.8 | 0.7 | 0.6 |
| NR_037675 |  | 0.034 | 0.8 | 0.7 | 0.6 |
| ENST00000612522 |  | 0.034 | 0.7 | 0.8 | 0.7 |
| NM_001039548 | KLHL35 | 0.034 | 0.6 | 0.6 | 0.5 |
| NR_126355 |  | 0.034 | 0.5 | 0.4 | 0.3 |
| AF034187 | STK38 | 0.034 | 0.5 | 0.4 | 0.3 |
| NM_182538 | SPNS3 | 0.034 | 0.6 | 0.6 | 0.5 |
| NR_120683 |  | 0.034 | 0.8 | 0.7 | 0.6 |
| ENST00000530028 | EID1 | 0.034 | 0.9 | 0.8 | 0.7 |
| NR_126522 |  | 0.034 | 0.6 | 0.5 | 0.4 |
| NM_001823 | CKB | 0.034 | 0.7 | 0.6 | 0.6 |
| ENST00000484368 | PIGC | 0.037 | 0.5 | 0.5 | 0.4 |
| NM_021255 | PELI2 | 0.037 | 0.4 | 0.5 | 0.4 |
| NM_001666 | ARHGAP4 | 0.037 | 0.6 | 0.5 | 0.4 |
| NM_016558 | SCAND1 | 0.037 | 0.8 | 0.7 | 0.6 |
| NM_005949 | MT1F | 0.037 | 0.7 | 0.7 | 0.5 |
| NM_032588 | TRIM63 | 0.037 | 0.5 | 0.4 | 0.3 |
| NM_001127192 | CNBP | 0.037 | 0.8 | 0.7 | 0.7 |
| NM_001317770 | MED29 | 0.037 | 0.7 | 0.7 | 0.6 |
| NM_181515 | MRPL21 | 0.037 | 0.7 | 0.8 | 0.7 |
| NM_020944 | GBA2 | 0.037 | 0.3 | 0.2 | 0.2 |
| NM_016061 | YPEL5 | 0.037 | 0.7 | 0.7 | 0.6 |
| NM_030615 | KIF25 | 0.037 | 0.4 | 0.5 | 0.2 |
| NM_130898 | CREB3L4 | 0.037 | 0.7 | 0.7 | 0.7 |
| NR_103768 |  | 0.037 | 0.7 | 0.6 | 0.5 |
| NM_014153 | ZC3H7A | 0.037 | 0.9 | 0.9 | 0.7 |
| NM_006388 | HTATIP | 0.037 | 0.6 | 0.6 | 0.5 |
| NM_145252 | ZG16B | 0.037 | 0.6 | 0.6 | 0.5 |
| NM_032349 | NUDT16L1 | 0.037 | 0.8 | 0.7 | 0.7 |
| ENST00000418623 | RPL22P1 | 0.041 | 0.8 | 0.8 | 0.7 |
| NM_138355 | SCRN2 | 0.041 | 0.7 | 0.7 | 0.5 |
| NR_120526 |  | 0.041 | 0.4 | 0.2 | 0.3 |
| NM_139015 | SPPL3 | 0.042 | 0.8 | 0.6 | 0.5 |
| NM_005078 | TLE3 | 0.043 | 0.8 | 0.8 | 0.7 |
| NM_014155 | ZBTB44 | 0.045 | 0.6 | 0.5 | 0.4 |
| NR_046283 |  | 0.045 | 0.6 | 0.6 | 0.5 |
| NM_012309 | SHANK2 | 0.045 | 0.7 | 0.7 | 0.5 |
| NM_001032374 | ZNF226 | 0.045 | 0.6 | 0.6 | 0.5 |
| NM_181531 | BTN2A2 | 0.045 | 0.7 | 0.7 | 0.6 |
| NM_138355 | SCRN2 | 0.046 | 0.7 | 0.6 | 0.4 |
| NM_024591 | CHMP6 | 0.046 | 0.8 | 0.8 | 0.6 |
| NM_145341 | PDCD4 | 0.046 | 0.7 | 0.7 | 0.5 |
| NM_001080417 | ZNF629 | 0.046 | 0.7 | 0.7 | 0.5 |
| NM_206827 | RASL11A | 0.046 | 0.5 | 0.5 | 0.4 |
| NM_015650 | TRAF3IP1 | 0.046 | 0.7 | 0.8 | 0.6 |
| NM_001040025 | ARL16 | 0.047 | 0.7 | 0.6 | 0.6 |
| NM_031452 | FAM103A1 | 0.047 | 0.8 | 0.8 | 0.8 |
| NM_020773 | TBC1D14 | 0.047 | 0.8 | 0.8 | 0.7 |
| NM_006286 | TFDP2 | 0.047 | 0.7 | 0.7 | 0.6 |
| NM_139250 | CTAG1A | 0.049 | 0.5 | 0.5 | 0.4 |
| NR_111907 |  | 0.049 | 0.6 | 0.5 | 0.4 |
| ENST00000660738 |  | 0.049 | 0.6 | 0.6 | 0.6 |
| NM_000400 | ERCC2 | 0.050 | 0.7 | 0.7 | 0.6 |
| NM_005119 | THRAP3 | 0.050 | 0.8 | 0.8 | 0.7 |
| NM_001007255 | KLHDC9 | 0.050 | 0.7 | 0.9 | 0.6 |
| NM_032752 | ZNF496 | 0.050 | 0.7 | 0.8 | 0.7 |

List of genes significantly downregulated (FDR-adjusted *p* < 0.05) in the A200-XT group compared with the NT group (n = 4 per group). Gene expression differences were identified by microarray analysis and FDR correction as described in the Methods section.
